# Biological Activities of Ethanolic Extracts from Deep-Sea Antarctic Marine Sponges

**DOI:** 10.3390/md11041126

**Published:** 2013-04-02

**Authors:** Tom Turk, Jerneja Ambrožič Avguštin, Urška Batista, Gašper Strugar, Rok Kosmina, Sandra Čivović, Dorte Janussen, Silke Kauferstein, Dietrich Mebs, Kristina Sepčić

**Affiliations:** 1 Department of Biology, Biotechnical Faculty, University of Ljubljana, Večna pot 111, 1000 Ljubljana, Slovenia; E-Mails: tom.turk@bf.uni-lj.si (T.T.); jerneja.ambrozic@bf.uni-lj.si (J.A.A.); gasper.strugar@gmail.com (G.S.); rkosmina@health.sdu.dk (R.K.); supernova.sandra@gmail.com (S.Č.); 2 Institute of Biophysics, Faculty of Medicine, University of Ljubljana, Lipičeva 2, 1000 Ljubljana, Slovenia; E-Mail: urska.batista@mf.uni-lj.si; 3 Senckenberg Research Institute and Natural History Museum, Senckenberganlage 25, D-60325 Frankfurt, Germany; E-Mail: dorte.janussen@senckenberg.de; 4 Institute of Legal Medicine, University of Frankfurt, Kennedyallee 104, 60596 Frankfurt, Germany; E-Mails: kauferstein@em.uni-frankfurt.de (S.K.); mebs@em.uni-frankfurt.de (D.M.)

**Keywords:** Antarctic marine sponges, hemolysis, antibacterial activity, acetylcholinesterase inhibition, cytotoxicity

## Abstract

We report on the screening of ethanolic extracts from 33 deep-sea Antarctic marine sponges for different biological activities. We monitored hemolysis, inhibition of acetylcholinesterase, cytotoxicity towards normal and transformed cells and growth inhibition of laboratory, commensal and clinically and ecologically relevant bacteria. The most prominent activities were associated with the extracts from sponges belonging to the genus *Latrunculia*, which show all of these activities. While most of these activities are associated to already known secondary metabolites, the extremely strong acetylcholinesterase inhibitory potential appears to be related to a compound unknown to date. Extracts from *Tetilla leptoderma*, *Bathydorus* cf. *spinosus*, *Xestospongia* sp., *Rossella* sp., *Rossella* cf. *racovitzae* and *Halichondria osculum* were hemolytic, with the last two also showing moderate cytotoxic potential. The antibacterial tests showed significantly greater activities of the extracts of these Antarctic sponges towards ecologically relevant bacteria from sea water and from Arctic ice. This indicates their ecological relevance for inhibition of bacterial microfouling.

## 1. Introduction

The marine environment covers around 70% of the Earth surface and has extremely high biodiversity. It is also a rich source of natural compounds that have previously unrecognized chemical structures and biological activities. Since 1974, when the first sponge-derived natural products become part of the pharmacopeia (e.g., cytarabine [Ara-C] and vidarabine [Ara-A]), marine natural products have gained much research interest [1,2]. So far, over 20,000 natural products have been isolated and identified from various marine organisms [3], and about a dozen natural marine-derived compounds and their analogs or derivatives are currently in different phases of clinical trials [1,2]. 

However, research and isolation of these compounds has been mainly directed towards organisms from temperate and tropical seas. Although the polar marine regions comprise a large portion of the total ocean area of the world, with Antarctica alone representing 10%, the difficulties associated with access to these regions has meant that only 3% of the marine natural products described today are derived from polar environments [4,5]. The harsh environmental conditions in the polar regions have for a long time been considered to have negative effects on biodiversity, interspecies competition and the incidence of chemical defense in polar marine organisms [4]. However, it was shown recently that the biodiversity in these areas can be compared to that in temperate and tropical regions, which applies especially to the marine sponges [4,6]. 

Sponges are sessile marine feeders and are the predominant species of the Antarctic benthos [7]. They have developed many adaptations that have allowed them to survive in cold waters, like the production of “antifreeze” peptides [8] and their ability to exploit additional nutrient resources [6]. Moreover, Antarctic sponges represent the main prey of various predators, like sea stars and nudibranchs, and they provide a habitat for associated fauna, which range from microorganisms, like bacteria and diatoms, to larger invertebrates, like crustaceans, bivalves and polychaetes [6,9,10]. It is therefore not surprising that marine sponges from polar environments have developed a collection of chemical defense mechanisms that are used as repellents and in territorial competition. For instance, a large number of organohalogens have been detected in Antarctic sponges, which might be released into the seawater by the producers and which eventually enter the food web, since some of these compounds were also discovered in marine mammals [11].

In general, sponge research is yielding more than 200 new pharmacologically active metabolites per year at present, and several compounds that are derived from sponges or from sponge-associated bacteria are in different phases of clinical trials. To shed some light on pharmacologically interesting novel compounds from polar environments in the present study, we report the screening results for hemolytic, anti-acetylcholinesterase (AChE) and cytotoxic activities detected in ethanolic extracts from 33 Antarctic marine sponge species. The extracts were also assayed against a broad range of laboratory, commensal and clinically relevant bacterial strains, and their potential ecological role was assessed by testing their inhibitory potential on ecologically relevant bacteria isolated from marine waters and from Arctic ice.

## 2. Results and Discussion

In contrast to our previous study on aqueous and organic extracts from tropical marine sponges, where we described at least one potent biological activity in each of the tested extract [12], ethanolic extracts from the Antarctic sponge species tested in the present study did not show such a wide spectrum of bioactivities. The sponge genus that showed the broadest spectrum and highest activities here was *Latrunculia* (*i.e*., extracts #37/L and #46). This sponge genus is known to produce numerous bioactive natural products, such as, for example, the cytotoxic discorhabdins [13,14] and the 2-thiazolidinone macrolides known as the latrunculins, which can disrupt microfilament organization [15].

Hemolytic activity was associated with only ten of the sponge samples and was most prominent in the extract of the sponge *Latrunculia* cf. *bocagei* (#46) (Table 1). However, it is interesting to note that the sponge extract from *Latrunculia* cf. *lendenfeldi* (#37/L) did not show any hemolytic activity. Although lower than the activity seen for extract #46, high hemolytic activity was detected in the extracts from *Bathydorus* cf. *spinosus* (#8), *Halichondria osculum* (#45h), *Xestospongia* sp. (#48/1), *Rossella* cf. *racovitzae* (#167), *Tetilla leptoderma* (#55), and from a sponge of the family of Microcionidae (#41). No previous data on hemolytic activities of the marine sponge extracts used in this study have been reported to date. In comparison, in our previous study on the biological activities of extracts from tropical marine sponges [12], hemolytic activity was present in about half of the organic extracts tested. 

AChE inhibitors are compounds that have potential use in treating disorders like Alzheimer disease, myasthenia gravis and glaucoma [16]. In the present study, AChE inhibitory activities were observed in two extracts only, which were from sponges of the same genus: *Latrunculia* cf. *lendenfeldi* (#37/L) and *Latrunculia* cf. *bocagei* (#46), (Table 1). The AChE-inhibitory potential in these extracts was extremely high; indeed, 50% of the enzyme activity could be inhibited by only few ng/mL, which indicates that the isolated active substance should probably act in the range of picograms. A hexane extract from the Red Sea *Latrunculia magnifica* was reported to contain a partly characterized compound that can induce inhibition of butyrylcholinesterase [17], although, to the best of our knowledge, the exact chemical structure of this active component was never determined. The observed AChE-inhibitory potential of *Latrunculia* extracts in our study is however not linked to latrunculins, biologically active secondary metabolites present in this sponge genus [15], since in the control experiment, latrunculin A (up to the concentration of 5 μg/mL) failed to inhibit AChE. Due to the very low amounts of any such active compound(s) in the extracts from *L.* cf. *lendenfeldi* (#37/L) and *L.* cf. *bocagei* (#46) that are needed for the inhibition of AChE, isolation of the active compound(s) from this sponge genus appears to be worth further study. 

**Table 1:** Hemolytic, anti-acetylcholinesterase and cytotoxic activities of the most active sponge extracts. Empty spaces in columns denote that the tested sponge extract exhibited no hemolytic or anti-acetylcholinesterase (AChE) activity.

Sponge Species	S#	Hemolytic Activity ^1^	Anti-AChE Activity ^2^	Cytotoxic Activity ^3^	
				V-79 cells	CaCo-2 cells
*Bathydorus* cf. *spinosus*	8	0.045		95.2 ± 7.7	91.8 ± 10.9
Unidentified sponge 1	10	0.014		97.6 ± 8.5	85.7 ± 8.1 **
*Cinachyra* cf. *barbata*	27	0.008		97.9 ± 11.2	95.0 ± 8.9
*Rossella* sp.	34	0.0025		101.1 ± 12.9	100.0 ± 10.0
*Latrunculia* cf. *lendenfeldi*	37/L		1.3	0	2.1 ± 1.6
Microcionidae spp.	41	0.017		102.8 ± 9.9	102.3 ± 11.2
*Halichondria osculum*	45h	0.025		62.9 ± 11.9	56.6 ± 6.7 *
*Latrunculia* cf. *bocagei*	46	0.15	9	0	0
*Xestospongia* sp.	48/1	0.015		89.0 ± 8.7	95.2 ± 11.7
*Isodictya toxophila*	51			84.7 ± 10.4	97.7 ± 8.9 **
*Tetilla leptoderma*	55	0.014		91.9 ± 11.4	96.7 ± 11.1
*Demospongia* sp.	124			102.2 ± 12.2	96.2 ± 11.0
*Rossella* cf. *racovitzae*	167	0.012		55.7 ± 11.9	93.5 ± 7.5 **

S#, sponge extract code; ^1^ expressed as 1/*t*_50_ (min^−1^) at 400 μg dried extract/mL in the assay; ^2^ expressed as concentration of the dried extract (ng/mL) that resulted in 50% inhibition of the enzyme activity; ^3^ viability of V-79 and CaCo-2 cell lines treated with 100 μg/mL of the dried extract, expressed as % of control; significant difference in cytotoxic activity between V-79 and CaCo-2 cell lines (* *p* < 0.05; ** *p* < 0.01).

The discovery of new antibiotics is one of the most important goals in biomedical research, as the appearance of multiresistant bacterial strains has made certain human and animal infections virtually untreatable. Sponges are known to contain a high number of compounds that act against terrestrial pathogenic bacteria, while considerably lower activities have been observed against marine bacteria [18]. Furthermore, in comparison with sponges found in temperate and tropical seas, Antarctic sponges have been reported to have a smaller number of antimicrobial secondary metabolites [19] that show generally weaker activities [20]. Previous screenings of crude extracts from 93 Arctic sponges against bacteria and fungi associated with opportunistic infections showed that about 10% of the sponges yielded significant antimicrobial activities, with IC_50_ values from 0.2 to 5 μg/mL [5]. In the present study, only eight ethanolic extracts from Antarctic sponges showed antimicrobial activities against laboratory, commensal and pathogenic bacteria (Table 2). The extracts from *Halichondria osculum* (#45h), *Hemigellius bidens* (#41a), *Latrunculia* cf. *lendenfeldi* (#37/L) and the sponge *Rossella* sp. (#4) were active against the majority of the bacterial strains tested, with a clear specificity towards Gram-positive bacteria. The highest activity and thus lowest MIC, was seen for the *L.* cf. *lendenfeldi* (#37/L) extract; e.g., its MIC for inhibition of *Staphylococcus aureus* (MRSA) S-943 was only 15 μg/mL. This sponge extract, albeit at approximately five-fold to 50-fold higher concentrations, also showed the greatest efficacy for the inhibition of the growth of Gram-negative bacteria, as mainly seen against the *Escherichia coli* strains. The activities of the remaining four sponge extracts (unidentified Demosponge [#38], *Isodictya setifera* [#58], *Myxilla* [#26] and *Haliclona flagellifera* [#40a]) were generally lower and seen only towards a few bacterial strains.

**Table 2:** Antibacterial activities (MICs) of the sponge extracts against the laboratory, commensal and clinically relevant bacterial strains. Empty spaces in columns denote that the tested sponge extract exhibited no antibacterial activity.

Bacterial Strain	Sponge Extract MIC (μg/mL)							
	*Latrunculia* cf*. lendenfeldi* (#37L)	Demosponge (#38)	*Halichondria osculum* (#45h)	*Isodictya setifera* (#58)	*Hemigellius bidens* (#41a)	*Rossella* sp. (#4)	*Myxilla* sp. (#26)	*Haliclona* (*Gellius*) *flagellifera* (#40a)
*Staphylococcus aureus* (MRSA) S-943 ^A^	15		200	600	400	350		
*S. pseudintermedius* (MRSP) S-053 ^A^	80		200		400			
*S. pseudintermedius* (MRSP) S-043 ^A^	80	50	200		400	30		
*Listeria monocytogenes*	90		250		500			
*Staphylococcus epidermidis* EXB-V55	100	150	250	200	80	300		
*Staphylococcus aureus* 10F	100		250		400	300		
*Bacillus subtilis* EXB-V68	8		2.5		80	30	60	65
*Macrococcus* 1F	10	7	4	100	6	200	400	650
*Micrococcus* 2F	70		25	150	60	150		
*Escherichia coli* HB101	700		250		400			
*Escherichia coli* EXB-V1	700				400			
ESBL- *E. coli* 206 (CTX-M-1; ST131) ^A^	750							
ESBL- *E. coli* 192 (CTX-M-9; ST131) ^A^	750							
ESBL- *E. coli* MS 30 (CTX-M-2) ^A^	700				500			
*Acinetobacter* 1C	70	400	250					
KPC- *Klebsiella pneumonia* ^A^								
*Enterobacter* EXB-V11								
*Pseudomonas aeruginosa* EXB-V28								
*Pseudomonas aeruginosa* 06131 ^A^	700				400			
*Pseudomonas aeruginosa* 8591 ^A^								

^A^ multiresistant isolate.

Our data are generally in line with those obtained by McClintock and Gauthier [20], who screened non-polar extracts of 17 Antarctic sponges for inhibitory activities against bacteria and fungi. They showed particularly strong inhibitory activities associated with extracts from the sponge species belonging to the genera *Latrunculia* and *Haliclona*. The antibacterial activity of the extract from *L.* cf. *lendenfeldi* might be related to the presence of *Latrunculia*-associated natural products that have already been reported to have inhibitory potential against various Gram-positive and Gram-negative bacteria, which are known as the discorhabdins [21,22,23] and trunculins [24]. Furthermore, sponges of the genus *Haliclona*, which also include polar species, are known to contain antibacterial 3-alkylpyridinium alkaloids [25,26]. Similarly, sesquiterpenoids halichonadins [27] and a galactoside-specific lectin [28] from *Halichondria* sponges have been described to have antibacterial and antifungal effects, while extracts from sponges of the genus *Hemigellius* have not yet been reported to show any antibiotic properties. However, the present study shows antibacterial activities of *Hemigellius bidens* extract against all of the tested Gram-positive bacterial strains and also against a clinical multiresistant *Pseudomonas aeruginosa* isolate. Furthermore, the same extract inhibited the growth of *E. coli* laboratory strains and, to a lesser extent, of an extended-spectrum β-lactamase (ESBL)-producing clinical isolate, although it was ineffective against the pandemic, virulent and multiresistant *E. coli* ST131 isolate. These data should be borne in mind when screening tests are performed using strains that are solely from nonclinical environments. Weak antimicrobial activities of extracts from sponges belonging to the Antarctic genera of *Isodictya* have also been reported [20,29]. Moreover, a *P. aeruginosa* strain that is associated with this sponge was shown to have alkaloids that can inhibit the growth of Gram-positive bacteria [30]. Antimicrobial substances that have been derived from sponge-associated bacteria have also been reported for several other sponge species [18], and these might be responsible in part for the observed antimicrobial activities. Screening of methanolic extracts of *Myxilla arenaria* from the Indian Ocean did not reveal any activity against pathogenic bacteria and fungi [31]. To the best of our knowledge, there are no other reports on antibacterial activities of this sponge genus. 

Further analyses of the antibacterial potential of the Antarctic sponge extracts in the present study revealed significantly higher activities against Gram-negative bacterial strains isolated from sea water and from Arctic ice (Table 3). All of the already mentioned antibacterial sponge extracts inhibited the growth of these marine bacteria, with the most sensitive species being *Pseudoalteromonas* sp. Again, as in the case of pathogenic bacteria, the lowest minimal inhibitory concentration (MIC) values against the majority of the strains tested were observed for the *Latrunculia* cf. *lendenfeldi* (#37/L) extract. The growth of *Pseudoalteromonas* sp. was moderately inhibited also by other sponge extracts, as mainly those from *Cinachyra* cf. *barbata* (#27), *Rossella* sp. (#34), and *Homaxinella* cf. *balfouriensis* (#52). *Janthinobacterium svalbardensis*, a bacterial strain isolated from Arctic ice, was also highly susceptible to the *L.* cf. *lendenfeldi* (#37/L) extract, with a MIC of 90 ng/mL. It is interesting to note that all three *Pseudomonas* strains isolated from Arctic ice were resistant to all of the sponge extracts tested, which was also the case for most of the pathogenic strains of these bacteria (Table 1). The higher antibacterial potential of sponge extracts towards ecologically relevant bacteria probably reflects the sponge defense mechanisms that are designed to prevent bacterial colonization. A similar conclusion was reached also in a previous screening test of polar sponge extracts on sympatric bacterial strains isolated from seawater, sediment and stones [19]. Surprisingly, lipophilic and hydrophilic extracts from Antarctic sponges have shown only mild inhibitory activities against sponge-associated bacteria, which indicates that symbiotic bacteria are probably not a common threat to sponges [29]; indeed, these symbiotic bacteria might instead be used as an alternative source of nutrients when these are scarce in the sponge environment [6]. 

Compounds that show selective cytotoxic activities might be candidates for new chemotherapeutics, which is still the main focus of natural product chemists and pharmaceutical companies [18]. Currently, reports on cytotoxic metabolites from sponges that live in the polar regions are scarce. In the present study, the most prominent cytotoxicity was detected in the extracts of *Latrunculia* cf. *lendenfeldi* (#37/L) and *Latrunculia* cf. *bocagei* (#46) (Table 1). This is not surprising, knowing that this sponge genus has a variety of cytotoxic pyrroloiminoquinone alkaloids, the discorhabdins [13,14], that act on various tumor cell lines. Moderate inhibition of cell growth was observed here also using the extracts from *Halichondria osculum* (#45h) and *Rossella* cf. *racovitzae* (#167). Sponges of the genus *Halichondria* from temperate waters have been reported to contain cytotoxic depsipeptides [32] and steroids [33], as well as the halichondrins, polyether macrolides that show potent cell inhibitory activities at nanomolar concentrations [34,35]. Regarding the sponges of the genus *Rossella*, we were not able to retrieve any data on their cytotoxic potential. There were no distinctive selective cytotoxicities seen against the transformed cell lines used in the present study (CaCo-2 cells) with any of the sponge extracts tested, except for non-identified sponge 1 (#10) and *Halichondria osculum* (#45h). The extracts from *Rossella* cf. *racovitzae* and *Isodictya toxophila* were even significantly more active against the normal cell line. 

**Table 3:** Antibacterial activities (minimal inhibitory concentrations (MICs)) of the sponge extracts against the environmental bacterial strains. Empty spaces in columns denote that the tested sponge extract exhibited no antibacterial activity.

Bacterial Strain	Sponge Extract MIC (μg/mL)							
	*Latrunculia* cf. *lendenfeldi* (#37L)	Demosponge (#38)	*Halichondria osculum* (#45h)	*Isodictya setifera* (#58)	*Hemigellius bidens* (#41a)	*Rossella* sp. (#4)	*Myxilla* sp. (#26)	*Haliclona (Gellius) flagellifera* (#40a)
*Exignuobacterium* sp. *	1.8	100	80	70	120	95	110	70
*Pseudoalteromonas* sp. *	0.36	90	65		110	100	90	70
*Alteromonas* sp. *	89.6	95	70		200	80	150	
*Vibrio ruber* *	0.9	70	85	65	120	90	90	
Janthinobacterium svalbardensis **	0.09	85	75		100	100	95	70
*Pseudomonas* CR 13 **								
*Pseudomonas* CR 14 **								
*Pseudomonas* CR 285 **								

* strains isolated from sea water; ** strains isolated from Arctic ice.

## 3. Experimental Section

### 3.1. Sponge Collection

Thirty-three sponge specimens that represented 28 species were collected in the Antarctic waters (60° to 70° S; 8° to 61° W) by bottom trawling and dredging at depths between 200 m and 900 m, during two cruises of the German Research Vessel “Polarstern” in 2006/07 and 2008. The specimens were identified to at least the family level (Supplementary Table S1), and they were immediately frozen and kept at −20 °C. The sponge samples were lyophilized prior to the extraction. 

### 3.2. Preparation of Extracts

The weights of the lyophilized sponge samples were in the range of 0.52 g to 3.78 g. The lyophilized material was macerated and placed into labelled glass tubes, and 10 mL 96% ethanol (Merck, Germany) was added to each tube. The tubes were sealed with metal stoppers and parafilm and were shaken overnight (600 rpm) at 37 °C. The extracts were then filtered, and the solvent was evaporated to the final volume of 1 mL. The dry weight of each sample was determined by drying an aliquot of a sample in a preweighed round-bottomed flask, with evaporation of the solvent under vacuum at 45 °C. The dry weight was expressed in mg/mL of the 1 mL extract volumes prepared. Stock concentrations were in the range of 3.1 mg/mL to 10.3 mg/mL. The sponge species and the dry weights of the extracts are given in Supplementary Table S1. 

### 3.3. Hemolytic Activity Assay

Fresh bovine erythrocytes were washed three times in physiological saline prior to use and then resuspended in erythrocyte buffer (130 mM NaCl, 20 mM TRIS-HCl, pH 7.4). The erythrocyte suspension had an initial absorption at 650 nm of 1.0 ± 0.01 AU. The hemolytic activity was assayed using a microplate VIS absorption reader (Dynex, USA), as described previously [12]. With 100 μL of erythrocyte buffer in each microplate well, the ethanolic sponge extracts were added to each well at different final dry-extract concentrations, followed by 100 μL erythrocyte suspension. The volume of ethanol in the final reaction mixture did not exceed 20%, a concentration that was tested and shown not to be lytic. The time course of hemolysis was immediately started and monitored for 30 min. The hemolytic activity was expressed as the half-time of hemolysis (*t*_50_); e.g., the time in which the apparent absorbance at 650 nm dropped from 0.5 to 0.25. All of these measurements were carried out in triplicates at 25 °C. The samples showing the highest hemolytic activities were further diluted with ethanol (1:10 and 1:100) for confirmation of the hemolytic activities in further assays. 

### 3.4. Antibacterial Activity Assay

The antibacterial activities of the sponge extracts were determined using the agar diffusion method, as described previously [12]. A broad range of bacterial strains was tested, which included ecologically relevant Gram-negative marine bacteria isolated from sea water (*Exignuobacterium* sp., *Pseudoalteromonas* sp., *Alteromonas* sp. and *Vibrio ruber*) and from Arctic ice (*Janthinobacterium svalbardensis*, *Pseudomonas* CR 13, *Pseudomonas* CR 14 and *Pseudomonas* CR 285). These antibacterial activities were also assayed against the following characterized strains of different origins: (1) laboratory strains: *E. coli* HB101, *Bacillus subtilis* EXB-V68, *Enterobacter* EXB-V11, *P. aeruginosa* EXB-V28 and *Staphylococcus epidermidis* EXB-V55; (2) commensal isolates from dog skin: *S. aureus* 10F, *Macrococcus* 1F, *Micrococcus* 2F and *Acinetobacter* 1C; (3) a food isolate: *Listeria monocytogenes*; and (4) clinical isolates: methicillin-resistant *S. aureus* (MRSA) S-943, methicillin-resistant *Staphylococcus pseudintermedius* (MRSP) S-053 and S-043 ESBL-producing *E. coli* 206 (CTX-M-1 group; ST131), ESBL-*E. coli* 192 (CTX-M-9; ST131), ESBL-*E. coli* 30 (CTX-M-2), carbapenemase producing (KPC) *Klebsiella pneumoniae*, *P. aeruginosa* 06131 and *P. aeruginosa* 8591. The strains were obtained from the EX (extremophilic microorganisms) and GM (genetic laboratory microbes) culture collections of the Chair of Molecular Genetics and Microbiology of the Biotechnical Faculty and of the Institute of Microbiology and Parasitology, Veterinary Faculty, University of Ljubljana, Slovenia. The precultured bacteria (laboratory, commensal and clinically relevant strains and strains isolated from Arctic ice) were grown in LB broth (Sigma, USA) and were used for the inoculation of Luria broth agar plates, to a final cell concentration of 5 × 10^8^/mL. Four holes of 1 cm in diameter were made in the agar of each agar plate, which were then filled with 100 μL of an ethanolic extract. Ethanol was tested for its antimicrobial activity (100 μL) as a control. The inhibition zone for each sample was determined after overnight incubation of the plates at 37 °C. The plates containing the bacteria isolated from Arctic ice were incubated at 22 °C. The extracts showing the highest inhibition of bacterial growth were further diluted with ethanol and used to determine the minimal inhibitory concentrations (MICs), which were defined as the lowest concentrations in μg/mL that inhibited the growth of tested microorganism 1 mm from the rim of the hole. All of the laboratory, commensal and clinical bacterial strains were also assayed with standard antibiotics (tetracycline, kanamycin, rifampicin, ampicillin and chloramphenicol; Supplementary Table S2). The marine bacteria were precultured in liquid medium prepared by dissolving 5 g peptone and 1 g yeast extract in 1 L of aqueous solution of MgCl_2_·6H_2_O (10 mM) and NaCl (300 mM). Agar plates containing this liquid medium were inoculated with the bacteria to a concentration of 5 × 10^8^/mL, and the antibacterial activities were tested as described above after an overnight incubation of the plates at 37 °C.

### 3.5. Acetylcholinesterase Inhibition Assay

The AChE inhibition assay was performed according to the method of Ellman *et al*. [36]. AChE from electric eel (Sigma, USA) was dissolved in 100 mM phosphate buffer (pH 7.4) to a concentration of 500 EU/mL. Prior to the assay, the enzyme was 100-fold diluted in the same buffer. With 100 μL Ellman reagent (5,5-dithiobis-2-nitrobenzoic acid) in 50 mM phosphate buffer (pH 7.4) containing the substrate acetylcholine (ACh) in 1 mM final concentration in each microplate well, 5 μL sponge extract and then 45 μL AChE were added to start the reaction. Ethanol (5 μL) was used as a control. Ethanol-dissolved latrunculin A (5 μL, Molecular probes, USA) was also assayed for its AChE-inhibitory potential in final concentrations from 0.005 to 5 μg/mL. The time course of the enzymatic reaction was monitored over 5 min at 405 nm and at 25 °C. All the measurements were performed in triplicate. The extracts that showed significant AChE inhibitory activity were further diluted (1:10 and 1:100) and tested for confirmation of the AChE inhibition activities in further assays. A VIS microplate reader (Dynex, USA) was used for these assays. 

### 3.6. Cytotoxic Activity

In contrast to the previous biological tests, the cytotoxic activity was assayed only on selected sponge extracts, as those from: *Bathydorus* cf*. spinosus* (#8), non-identified sponge 1(#10), *Cinachyra* cf. *barbata* (#27), *Rossella* sp. (#34), *Latrunculia* cf. *lendenfeldi* (#37/L), Microcionidae spp. (#41), *Halichondria osculum* (#45h), *Latrunculia* cf. *bocagei* (#46), *Xestospongia* (#48/1), *Isodictya toxophila* (#51), *Tetilla leptoderma* (#55), Demospongiae (#124) and *Rossella* cf. *racovitzae* (#167). The cell lines used were: V-79-379 A (V-79) cells (diploid lung fibroblasts from Chinese hamster) and CaCo-2 cells (human colon adenocarcinoma). The V-79 cells were grown in advanced Eagle’s minimal essential medium (Gibco, Invitrogen, UK) and the CaCo-2 cells in advanced RPMI 1640 (Gibco), both at 37 °C in a CO_2_ incubator (5% CO_2_, 95% air, 95% relative humidity). Both of these culture media were supplemented with 2 mmol/L l-glutamine (Gibco), 100 U/mL penicillin (Gibco), 100 μg/mL streptomycin (Gibco) and 5% fetal bovine serum (Gibco). For the *in vitro* cytotoxicity assay, the cells were plated in 96-well microtiter plates (100 μL, Costar, USA) at a concentration of 5000 cells/well (V-79 cells) and 10,000 cells/well (CaCo-2 cells). After a 3-h incubation, the ethanol-dissolved extracts prepared in the respective media without serum were added, to a final concentration of 0.1 mg/mL, and the incubations were carried out for 1 h (under cell growth conditions). Ethanol (20 μL) was used as a control. The cells were then washed once with medium, and fresh medium with fetal bovine serum was added for a further 48 h (under cell growth conditions). The cytotoxicity was determined using the MTS (=3-(4,5-dimethylthiazol-2-yl)-5-(3-carboxymethoxyphenyl)-2-(4-sulfophenyl)-2*H*-tetrazolium) test. To each well, 20 μL of MTS (CellTiter 96 AQueous Reagent, Promega, USA) was added to the cell culture. After 1 h, the absorbance at 490 nm was measured using a Bio-Tek microplate reader (Bio-Tek Instruments Inc., USA). The absorption corresponded to the amount of the soluble formazan product that was formed, which is directly proportional to the number of viable cells. The viability was calculated as the ratio between absorbance at 490 nm of the treated and control cells, expressed as a percentage. The data are presented by means ± SD of 3 independent experiments. The differences were analyzed using Student’s *t*-tests on two populations, with *p* < 0.05 and *p* < 0.01 considered significant.

## 4. Conclusions

The data from the present study are in line with previous reports on biological activities of polar marine sponges. Although the biological activities of these sponges are in generally associated with a lower number of species as compared to sponges from temperate and tropical regions, they are still widely unexplored and might provide valuable resources for new pharmaceutical lead compounds. One such example is seen with the extracts of the sponges of the genus *Latrunculia*, which were shown to contain an extremely potent inhibitor of AChE. 

## Supplementary Files

Supplementary File 1 Supplementary Information (PDF, 56 KB)
