# Supplementary material for: Biological Activities of Ethanolic Extracts from Deep-Sea Antarctic Marine Sponges
Source: Mar Drugs. 2013 Apr 2;11(4):1126–39. doi: 10.3390/md11041126 (PMC3705393; doi:10.3390/md11041126)
Supplement: Supplementary File 1 — Supplementary Information (PDF, 56 KB) [file marinedrugs-11-01126-s001.pdf]

Supplementary Information

Table S1. The sponge species used in this study, and the dry weights of their ethanolic extracts.

Sponge species	S#	Extract dry weight (mg/mL)
Rossella cf. *nuda/vanhoeffeni*	3	3.8
Rossella sp.	4	4.7
Tethyopsis sp.	6	4.6
Bathydorus spinosus	8	3.6
non-identified sponge 1	10	3.3
Myxilla sp.	26	7.4
Chinachyra cf. *barbata*	27	3.5
Rossella sp.	34	6.5
Demospongiae spp.	36	3.7
Rossella ractovitzae Topsent	37/R	5.7
Latrunculia cf. *lendenfeldi*	37/L	9
Demospongiae spp.	38	5.4
Haliclona (*Gellius*) *flagellifera*	40a	7.2
Hemigellius bidens	41a	5.9
Microcionidae spp.	41	4.5
Rossella cf. *racovitzae*	43	6.4
Halichondria osculum	45h	3.1
Demospongiae sp.	45d	3.9
Lantrunculia cf. *bocagei*	46	7.2
Xestospongia sp.	48/1	4
non-identified sponge 2	48/2	6.9
Isodictya toxophila	51	4.3
Homaxinella balfouriensis	52	4.6
Tetilla leptoderma	55	4.2
Haliclona flagellifera	56	5.3
Isodictya setifera	58	6.5
Suberitidae gen. sp.	63	4.2
Demospongiae sp.	105	4.9
Tetilla leptoderma	119	10.3
Demospongiae sp.	124	5
Rossella cf. *nuda/vanhoeffeni*	132	6.1
Rossella cf. *racovitzae*	166	6.5
Rossella cf. *racovitzae*	167	3.8

S#: sponge extract code.

Table S2. Antibacterial activities (MICs) of selected antibiotics against the laboratory, commensal and clinically relevant bacteria strains.

**Bacterial strain**	**Antibiotic MIC (µg/mL)**				
	**Tetracycline**	**Chloramphenicol**	**Kanamycin**	**Ampicillin**	**Rifampicin**
Staphylococcus aureus (MRSA) S-943 [A]	80	7	9	700	0.005
S. pseudintermedius (MRSP) S-053 [A]	2	500	250	3000	0.05
S. pseudintermedius (MRSP) S-043 [A]	50	8	250	700	0.06
Listeria monocytogenes	80	5	2.5	250	0.008
Staphylococcus epidermidis EXB-V55	0.2	4	0.4	0.8	0.02
Staphylococcus aureus 10F	2	20	8	0.7	0.5
Bacillus subtilis EXB-V68	0.03	0.8	8	2	0.07
Macrococcus 1F	0.15	9	0.09	0.5	0.007
Micrococcus 2F	0.2	2	8	0.5	0.009
Escherichia coli HB101	0.5	5	80	70	90
Escherichia coli EXB-V1	2	0.9	6	2000	8
ESBL-*E. coli* 206 (CTX-M-1; ST131) [A]	9	4	90	500	8
ESBL-*E. coli* 192 (CTX-M-9; ST131) [A]	0.5	3	9	5000	20
ESBL-*E. coli* MS 30 (CTX-M-2) [A]	9	0.9	7	>10,000	20
Acinetobacter 1C	0.05	9	200	5000	0.2
KPC-*Klebsiella pneumoniae* [A]	4	900	900	>10,000	70
Enterobacter EXB-V11	4	7	70	200	70
Pseudomonas aeruginosa EXB-V28	9	0.9	80	500	60
Pseudomonas aeruginosa 06131 [A]	20	7	60	200	15
Pseudomonas aeruginosa 8591 [A]	9	90	80	5000	60

A: multiresistant isolate.
